# Practice Guidelines for Clinical Pharmacists in Middle to Low Income Countries

**DOI:** 10.3389/fphar.2020.00978

**Published:** 2020-06-30

**Authors:** Elmien Bronkhorst, Andries G. S. Gous, Natalie Schellack

**Affiliations:** School of Pharmacy, Sefako Makgatho Health Sciences University, Pretoria, South Africa

**Keywords:** clinical pharmacy, pharmaceutical care, key-performance indicators, outcome measures, national drug policy

## Abstract

The profession of pharmacy is maturing as a clinical profession in South Africa and has experienced significant development over the past 10 years. The development of clinical pharmacy in Southern Africa started in the late 1980s. The Director-General of Health and Welfare requested an expansion of the pharmacist’s role in Southern Africa, in 1988, when he challenged pharmacists to be “more than just dispensers.” South Africa experience human resource challenges in terms of healthcare service delivery and the shortage of pharmacists has been acknowledged. Due to the human resource shortage, it is very difficult to allocate pharmacists to work in a clinical unit on a daily basis. This document serves to set out practice guidelines for clinical pharmacy in South Africa, and to indicate areas where clinical pharmacist should concentrate to build practice.

## Introduction

The profession of pharmacy is maturing as a clinical profession in South Africa and has experienced significant development and growth over the past 10 years. It is presently well positioned to transform itself from a product-orientated (including procurement, preparation and evaluation of drug products), to a patient-oriented profession (Hepler and Strand, 1990). The clinical pharmacist must fulfill an important role in the National Health Insurance (NHI). This strategic document represents a comprehensive narrative on the role of the clinical pharmacist in UHC through NHI for South Africa.

The American College of Clinical Pharmacy (ACCP) in 2006 defined the primary difference between clinical pharmacists and general registered pharmacists by clinical pharmacists’ ability to interact with patients and the fact that they can recommend specific drugs and patient specific drug dosages to improve patient well-being (Hepler, 2004). From clinical pharmacy, the term pharmaceutical care was born. The two concepts are compatible and seem to have similar goals. One way to distinguish between the two can be to describe clinical pharmacy as a practice of pharmacy within a larger pharmaceutical-care system, where the pharmacist will contribute. The goal is to achieve pharmacotherapeutic and quality-of-life patient outcomes (Murphy et al., 2006 **).** Pharmaceutical care can be defined as “the direct, responsible provision of medication-related care for achieving definite outcomes, which improve a patient’s quality of life” (American Society of Hospital Pharmacists, 1992). Thus, pharmaceutical care can be considered as a form of clinical pharmacy.

The development of clinical pharmacy in Southern Africa started in the late 1980s. In 1987, Dowse and Kanfer (1987) wrote of “the non-existence of clinical pharmacy in South Africa”. An expansion of the pharmacist’s role in Southern Africa was strongly requested by the Director-General of Health and Welfare in 1988, when he challenged pharmacists to be “more than just dispensers” (Summers, 1991). Toward the end of the millennium, a partial success was achieved by the pharmacy profession toward extending its role, which remained limited to underserved areas and met with opposition from the medical profession, particularly relating to the pharmacist’s ability to prescribe (Gilbert, 1998). However, although curricula have been adapted to prepare pharmacists for this new role, (Berg, 2001; Summers et al., 2001) developments in practice focused on other issues, such as the emerging HIV epidemic, which brought about substantial changes in the health care sector with regard to practice as well as legislation.

The process of pharmacists joining ward rounds to monitoring medication in South Africa, has already been explored by Summers (1991). She was of the opinion that clinical pharmacy should be regarded as a particular professional approach to hospital pharmacy. Summers (1991) noted that it is essential for pharmacists to see the full patient picture in order for them to evaluate drug therapy, and communicate effectively with other members of the health care team. Pharmacists needs to build good relationships and enable communication with the multidisciplinary healthcare team, requiring them to move out of the dispensary into the wards where drugs are administered and doctors’ rounds are done. Human resource challenges and a lack of trained clinical pharmacists resulted in low pharmacist presence in a clinical capacity. In particular, a lack of the following pharmaceutical care functions was identified in South-Africa, at that time:

- Therapeutic drug monitoring
- Adverse drug reaction monitoring
- In-service training for the nursing and medical staff
- Providing drug information
- Monitoring drug usage

During the early 1990’s, the South African Pharmacy Council called for a shift to a more professional health-care service from pharmacists, based on specific expertise of the pharmacists. The SAPC used the terms “primary care drug therapy” or “pharmacotherapy” to potentiate this expanded role (Gilbert, 1998).

The concept of pharmaceutical care has evolved into “comprehensive medication management” as part of clinical pharmacy. Medication management has expanded as a result of medication regimens becoming more complex and specialized, particularly in more complex patients, who may have as many as five comorbidities and take an average of eight medications concurrently. To achieve better outcomes with medication-therapies in such patients, the systematic and comprehensive management of medications is necessary.

South Africa face challenges regarding human resources in terms of healthcare service delivery (Matshotyana, 2009). A shortage of skilled personnel in key areas of the health sector exist and the shortage of pharmacists has been acknowledged. Pharmacists are also unevenly distributed between the public and private sector, with the public sector struggling, despite the introduction of various incentives, to attract and retain pharmacists (Padarath et al., 2003).

According to Schellack and Gous (2010) for a pharmacist to play an effective part in the multidisciplinary health care team, the pharmacist must be stationed in the wards when drugs are prescribed and administered. Doctors’ ward rounds can be considered an effective way of communication and a good training ground for ward pharmacists, although time-consuming. Combined with the low human resources of pharmacists, sub-optimal use or lack of technical support staff and the limitation of trained clinical pharmacists, have resulted in low pharmacist presence in the wards in a clinical capacity. In the current limited situation, the pharmacist could choose to focus on only specific functions (e.g. patient care), and once acquainted with these services, less time would be require to achieve similar outcomes.

Due to the human resource shortage, it is very difficult to allocate pharmacists to work in a clinical unit on a daily basis. This document serves to set out practice guidelines for clinical pharmacy in South Africa.

## Policy Options and Implications

### Definitions and Qualifiers

The objective is to advocate the rational and appropriate use of medicines through the practice of clinical pharmacy and pharmaceutical care, in the interest of promoting health and well-being for the people of South Africa. All activities directly or indirectly, should be patient-oriented.

A secondary objective is to standardize the quality of pharmaceutical care offered by pharmacists across different institutions in both the public and private healthcare sector in South Africa.

**Figure 1:**
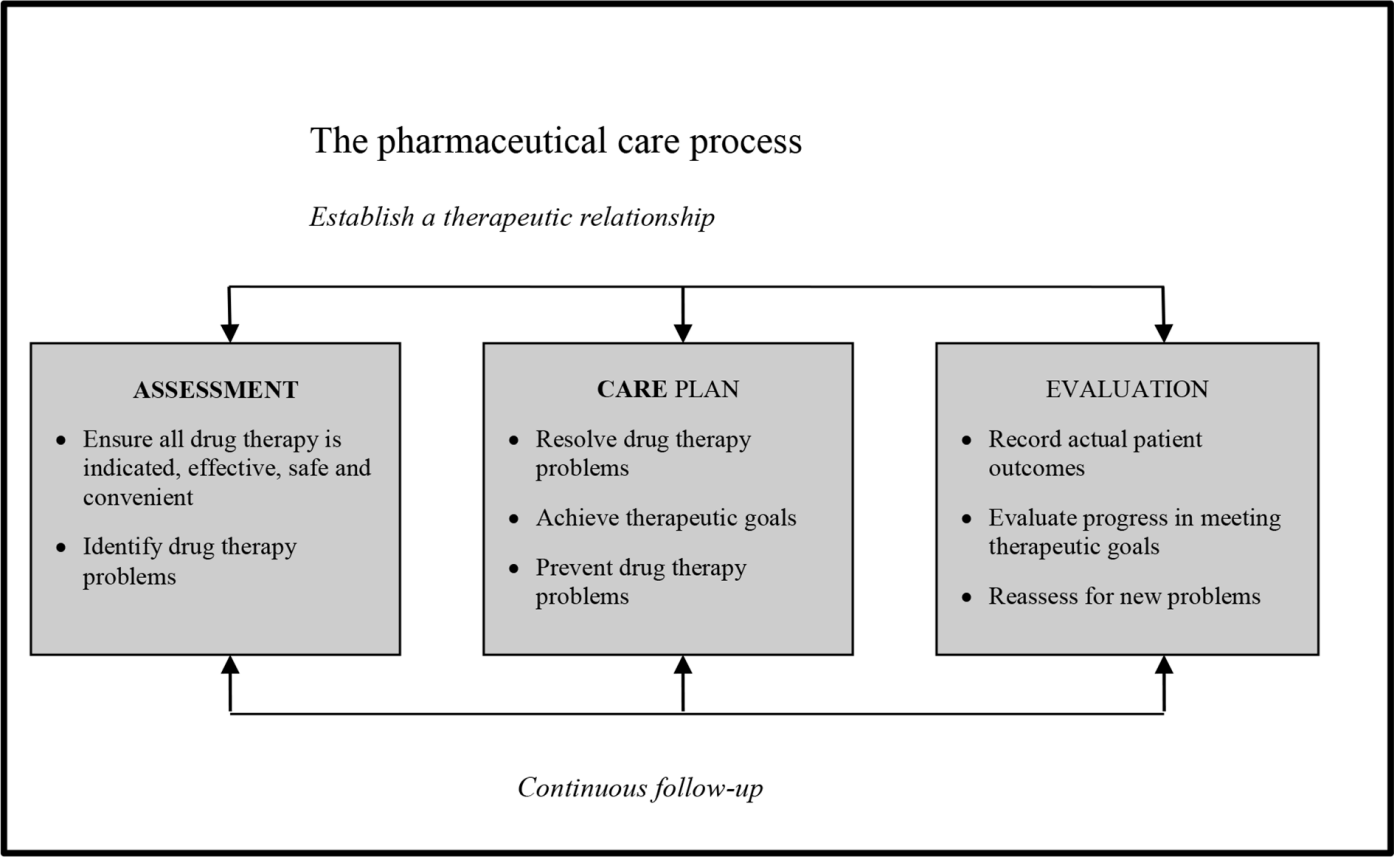
The Pharmaceutical Care Process.

“Clinical pharmacy is an area of pharmacy involved with the science, practice, activity and service to develop and promote the rational and appropriate use of medicines and pharmaceutical care, in the interest of the patient and the community”. During the Hoechst-Marion-Roussel lecture at the School of Pharmacy at the John Moores University in Liverpool, Strand explained that practice in pharmaceutical care has to be built up on one patient at a time, by first assessing the patient’s needs; developing a care plan and then monitoring the plan (Schellack and Gous, 2010). This process is depicted in **Figure 1**.

The practice guidelines will outline the process, as perceived in the South African context of:

- Assessment: Drug therapy evaluation and the process of identifying drug therapy problems
- Patient Care Plan Development, while resolving, identifying and preventing drug therapy problems

Evaluating and monitoring the Patient Care Plan and recording and evaluating the actual patient outcomes and reassess for new problems

### Extent and Operation

For clinical pharmacy to succeed in South Africa, it should be supported by the statutory body, management structures within the workplace, professional societies and funding structures.

Specially trained pharmacists in the area of clinical pharmacy to practize in direct patient care environments (American College of Clinical Pharmacy (ACCP), 2014) should deliver the practice of clinical pharmacy. These pharmacists should be registered at the statutory body as a specialist pharmacist after taking a special board certification exam and should take responsibility for continued learning. The clinical pharmacist should participate in indivualised patient care, taking responsibility for the medicine related health care needs of the patient. Patient care should be delivered in line with the National Health Insurance of South Africa and Standard Treatment Guidelines.

Clinical pharmacy is **NOT** restricted to hospital pharmacy or community based pharmacy. Hospital pharmacy should maintain good communication structures to support the health care needs of the patient.

### Core Competencies

According to the ACCP (American College of Clinical Pharmacy (ACCP), 2017), the expectation exists for a clinical pharmacist to be competent in six essential domains, including direct patient care, pharmacotherapy knowledge, systems-based care and population health, communication, professionalism and continuing professional development.

#### Direct Patient Care

Direct patient care can be defined as “the direct observation and evaluation of the patient’s medication-related needs and include the introduction of new medication, modification or discontinuation of existing therapy as well as the ongoing monitoring of patient outcomes in cooperation with other health professionals”. It involves specific activities to improve pharmacotherapy (Hepler, 2004).

#### Comprehensive Medicine Management

Comprehensive medication management is defined as: “the standard of care that ensures every patient’s medication (including prescription, nonprescription, *alternative, traditional, vitamins, or nutritional supplements) are individually assessed to determine whether each medication is appropriate for the patient, effective and indicated for the medical condition, safe given the comorbidities and other medications taken simultaneously, and able to be taken by the patient as intended*”(American College of Clinical Pharmacy (ACCP), 2017). Comprehensive medication management implies an individualized care plan formulated to achieve the intended goals of therapy. Appropriate knowledge of pharmacotherapeutic aspects are essential. Actual patient outcomes will be determined with appropriate follow-up. Optimizing each patient’s medication experience will occur, since the patient understands, agrees with, and actively participates in the treatment regimen.

#### Individualized Patient Care

Clinical pharmacists in South Africa, adopts the following set of pharmaceutical care medication-evaluation interventions, as developed by the American Society of Hospital Pharmacists (American Society of Hospital Pharmacists, 1992) to evaluate drug therapy. The form consists of the Pharmacist’s Patient Database Form, Current Drug Therapy Form, Laboratory Information, Drug Therapy Problem List, Drug Therapy Assessment Worksheet and Pharmacist’s Care Plan Monitoring Worksheet.

These activities will serve to optimize the use of medicines, decrease potential drug interactions and side effects, and monitor therapeutic outcomes. **Table 1** depicts a summary on evaluation of medication usage.

**Table 1:** Evaluation of medication usage.

Function	Explanation	Monitoring/Outcome measure
**Lack of correlation between drug therapy and medical problems**	Drugs without medical indications, unidentified medications, or untreated medical conditions, including any which required drug therapy.	Drugs discontinued, or introduced Unidentified medication destroyed
**Inappropriate drug selection**	Comparative efficacy and safety, and appropriateness for the individual patient	Limit side-effects or adverse drug reactions Optimize dose to reduce adverse drug reactions
**Drug regimen**	Inappropriate dose, dosing frequency, dosage form, route of administration (considering efficacy, safety, and convenience), or duration of therapy	Optimize dosing regimen including dose frequency, form, and route of administration Ensure adherence to therapy Avoid prolonged therapy
**Therapeutic duplication**	Treatment of any conditions with more types of medication than necessary.	Improved patient outcomes with reduced adverse drug effects
**Drug allergy or intolerance**	To any medicines, and method used to alert health care providers to the allergy/intolerance.	Avoided hypersensitivity reaction Ensure health care providers aware of allergy
**Adverse drug events**	Any possibly drug-related symptoms or medical problems, and the likelihood that the problem was drug related	Identify and stop offending medicine Report ADR on pharmacovigilance form
**Interactions**	Drug-drug interactions, drug-disease interactions, drug-nutrient interactions, and drug-laboratory interactions	Identify interaction and discontinue/replace identified drug Reduce adverse drug reaction
**Social or recreational drug use**	Smoking or alcohol Recreational drugs	Identify problem caused by social drug use Recommend optimal management
**Failure to receive therapy**	Reasons such as system errors or any other factors that could hinder achievement of therapeutic efficacy	Ensure availability of medicine supply to patients Address other factors or system errors

#### Professionalism and Continuous Professional Development

Clinical pharmacists are required to uphold the highest standards of integrity and honesty, always working in the best interest of patients. They have to commit to lifelong learning, self-assessment, and self-development, as well as to provide professional education to other healthcare professionals (Saseen et al., 2017).

#### Systems-Based Care and Population Health

Clinical pharmacists must use health delivery systems to optimize care of individual patients and participate to develop processes to improve medication use, applying knowledge of pharmacoeconomics (Saseen et al., 2017). They are required to partake in policy-writing as well as implementing and evaluating existing policies.

#### Communication

Clinical pharmacists are required to communicate effectively with patients, caregivers, and other health professionals, providing clear, concise consultations, in a language appropriate to the level of understanding of their audience (Saseen et al., 2017).

### Clinical Pharmacy and the National Health Insurance

In 2015, the National Department of Health (NDoH) published the White paper on National Health Insurance (NHI), toward universal health coverage (UHC) (National Department of Health, 2015). The NHI aims to provide all South Africans with affordable, personal health services. The services will be based on health needs, irrespective of their socio-economic status. The NHI further aims for improvement by increasing patients’ participation in managing illness, include addressing access to health care, reducing underlying causes of illness, and include addressing access to health care ensuring appropriate use of health care services and reducing health care-related errors or adverse events. Quality health care can be achieved by identifying discernible problems.

Furthermore, the NDoH published their Quality Standards for Healthcare Establishments in South Africa in 2011, (National Department of Health, 2011) which further discusses the global development of quality improvement for healthcare facilities. Pharmacists are required fulfill their role in the domain patient safety, clinical governance and care by reducing adverse events caused by medication or medication errors, as well as ensuring that medicines are available to patients (National Department of Health, 2011).

Clinical pharmacists should support the goals of the NHI (2015) aligning themselves with the National Core Standards to play a crucial role in the aim of NHI.

#### Health Objectives

Clinical pharmacists should participate in procuring and supplying essential drugs to all citizens and should subscribe to the policy of the Standard Treatment Guidelines as pertaining to Public Health Care. According to the National Drug Policy, essential drugs are described as drugs that treat the majority of conditions that are prevalent in the country and have been selected by an Essential Drug Selection Committee as the most cost-effective. Clinical pharmacists should partake in the selection of these committees.

#### Economic Objectives

Clinical pharmacists commit to partake in lowering the costs of drug use in both the private and public sector by promoting rational drug use and cost effective drug choices. The use of scarce resources can be optimized by promoting the establishment of partnerships between the private sector and the public sector, especially within the light of National Health Insurance.

#### National Development Objectives

Establishing clinical pharmacy practice as new area of specialization in South Africa will be accomplished through:

- Providing leadership and expertise in the field
- Hosting regular symposia, workshops and an annual conference
- Membership of the society which provides access to informative monthly branch meetings and society driven activities
- Lobbying regulatory bodies, health care funders and service providers
- Young and upcoming clinical pharmacists will be developed and supported
- Provide support to practicing clinical pharmacists

### Training and Education in Clinical Pharmacy

#### Aim and Objectives

##### Aim

To partake in training, advanced-level clinical pharmacists should be able to register at the South African Pharmacy Council as specialists, contribute to new knowledge in the field of clinical pharmacy and create specialists in the field of pharmacy for the development of health care in South Africa. Clinical pharmacists should participate in training of undergraduate pharmacists, in association with the Institution at which the undergraduate pharmacists are training. A mentorship program should be established between clinical pharmacists and the undergraduate pharmacist, but also to mentor new qualified clinical pharmacists.

##### Proposed Vision

To establish and coordinate the provision of pharmaceutical care and support for post graduate and independent research in clinical pharmacy, in order to increase the post graduate through-put and publications. This could also serve as a coordinating and collaborating platform for interdisciplinary research within and between the private and public sector.

##### Proposed Objectives

The specific objectives are as follows:

###### Teaching, Training and Service Delivery

- To facilitate and strengthen teaching and supervision of postgraduate students between the Private Health Care sector and academia.
- To teach, train and supervise postgraduate students at Post Graduate Diploma, Master’s, and Doctoral level in clinical pharmacy.
- To coordinate the teaching of research and clinical training within the clinical environment and provide community-based service training.
- To provide training in the form of workshops, symposia or short courses to staff by the clinical pharmacists in the hospital and clinic environment.
- To support clinical pharmacy as a learning component in undergraduate teaching.
- To achieve these competencies, clinical pharmacists must be prepared to complete the education and training and must commit to continued professional education to maintain competence through ongoing professional development.

###### Research

- To establish and maintain interdisciplinary and collaborative research projects to address relevant problems of healthcare needs in South Africa
- To produce research outputs (as articles, reports and congress presentations) of internationally recognized quality in clinical pharmacy
- To develop young researchers in clinical pharmacy, with particular emphasis on future clinical pharmacists
- To provide an interdisciplinary forum at which researchers can present protocols, progress reports and research reports to colleagues and receive feedback in a supportive environment

#### Proposed Levels of Training

The various levels of development during training will be assessed using competency standards developed in the UK. The General Level Framework (initially developed in the UK 2003), will be adapted for the South African Training and Practice Environment.

These tools will be utilized for assessment of the competence of a pharmacist and to identify areas for development, in the various stages of training. A peer, who will typically be a more experienced/senior clinical pharmacist, will do the assessment. The assessor has to be validated by another assessor/and or the South African Pharmacy Council.

The aim for clinical pharmacy in South Africa and other African countries is to promote advanced practice:

*“Practice that is so significantly different from that achieved at initial registration, after an initial degree, can be regarded advanced practice. Advanced practice will warrant recognition by professional peers as well as the public. The education, training and expertise of the practitioner ensures the capability of the advanced practitioner”.*

Advanced level pharmacists that are able to deliver specialized practice should be supported by relevant societies.

*“The specialised clinical discipline in which one practices, for example paediatrics refers to specialised”.*

**Figure 2:**
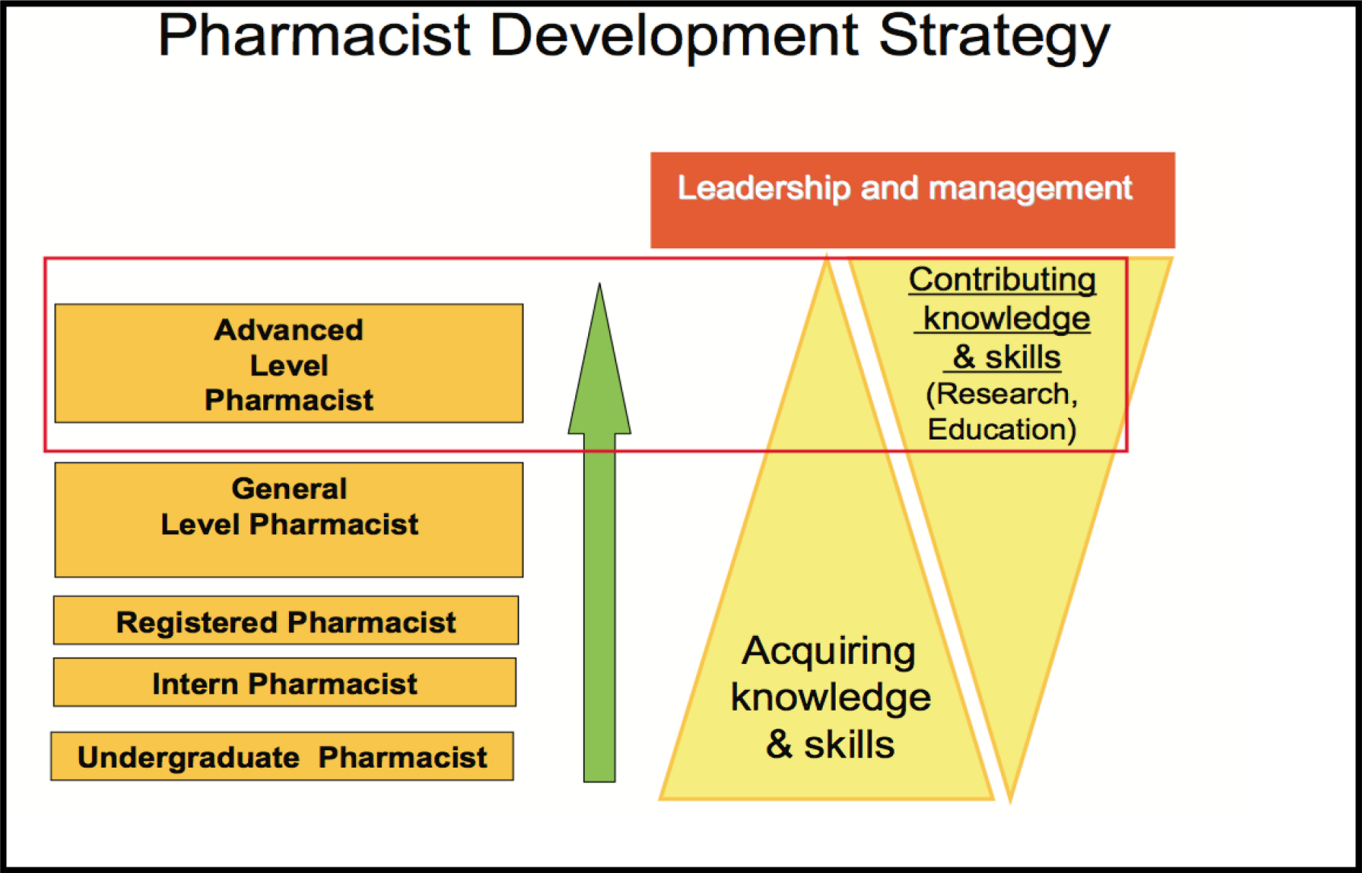
Development of advanced level pharmacists.

Currently, in the South African setting, there is a gap between general pharmacists and advanced level pharmacists for the general hospital/retail pharmacist interested in clinical pharmacy, but not necessarily at an advanced level. The society supports the development of a post-graduate certificate in clinical pharmacy, that can be developed using distance based education. The general level pharmacist (with a BPharm degree or equivalent) will be able to enter into this program, either to enroll in all modules to obtain the certificate, or only one module to receive a continuous professional development (CPD) certificate. See **Figure 2** for the development of advanced level pharmacists in South Africa.

The South African Pharmacy Council, as well as the Department of Health, will determine registration of a clinical pharmacist in South Africa and the qualifications necessary. However, the South African Society of Clinical Pharmacy proposes to support the idea that a clinical pharmacist can only practice with a relevant postgraduate Master’s degree or higher degree in clinical pharmacy. The idea of a board certification or pre-registration examination will also be supported.

## Barriers and Limitations to Implementation of Clinical Pharmacy Services

The top barriers to pharmaceutical care provision perceived by pharmacists include inconvenient access to patient medical information and a lack of staff and time constraints. The limited human resources in South Africa are one of the limitations of implementing clinical pharmacy services in hospitals. Pharmacists also expressed concern over not learning to communicate effectively with medical professionals. They have concerns regarding a lack of confidence to talk to medical professionals without undermining their professional judgment, and felt that their role was not valued by other health professions without any appreciation of their role within the health care team. Since the profession of clinical pharmacy is still a novel concept in South Africa, the lack of mentorship make this barriers more pronounced. With clear outcome measures and adequate education, these barriers can be overcome.

## Actionable Recommendation

### Documenting Clinical Pharmacy Interventions

Documentation of interventions and recommendations should be dictated by evidence-based practice and should only be done to promote and contribute to the welfare of the patient.

South Africa as a healthcare setting is diverse and the pharmacist providing services broadly classified and divided into the following must be documented:

- Patient related demographical details – for the pharmacist database
- Medication related consumption – for the pharmacist database and to monitor adherence and rational prescribing
- Laboratory values – to monitor disease progression and medication related side effects for the pharmacist database and monitoring parameters
- To assess drug therapy on a daily base according to the eleven categories
- Monitoring outcomes should be part of the clinical pharmacist’s initial assessment and clear pharmaceutical goals should be set

Documenting the interventions should be patient centered and should be supportive of the health care of the patient. It should be done according to Good Pharmacy Practice Guidelines (South African Pharmacy Council (SAPC), 2010) of the South African Pharmacy Council and the Medicine and Related Substances Act 101 of 1965.

#### Patient Medical Record

The clinical pharmacist makes regular interventions and can record it in the patients’ medical record. This should serve to supplement verbal communication as part of the health care team. Written communication is a permanent record in a legal document and where possible should not replace verbal communication. Verbal communication also serves to strengthen interdisciplinary relationships and should be promoted where possible.

When an entry is made into the medical record, the pharmacist should include the following information:

- The pharmacist should identify themselves and their discipline, with their degree and Council registration number, designation and contact details
- The instructions should be clearly written based on evidence based medicine with set pharmaceutical goals
- Date and time
- The inscription should follow a logical sequence e.g. the SOAP method (subjective patient details, objective clinical findings, assessment of the clinical problem and the proposed plan with the pharmacy goals

The following should also be taken into consideration:

- The clinical pharmacist should note recommendations to allow room for discussion within the health care team.
- Recognized medical abbreviations should be used
- The medication related action plan should be noted with clear goals
- Any relevant discussions that have taken place surrounding the issue should be noted
- All interventions should be signed and dated

A guideline for activities that can be documented by the clinical pharmacist is provided in **Table 2**.

**Table 2:** Guidelines for the documentation of clinical activities.

Clinical Activity	Activity Description	Data Collection
Patient information and medical history	This includes all the administrative and general information that can be obtained by an interview with the patient or from medical records in the instance of neonates or sedated/disorientated patients. Other information that can be included is the vital signs on admission, the history of the present illness, the past medical history of surgery, medication used before admission at the hospital, family and social history, general lifestyle, acute and chronic medical problems, social drug use, and allergies. This can also be used to see if the present illness could be a result of an adverse effect of the patient's demographics.	**Per patient** and is completed on the first visit to the patient.
Current drug therapy and adverse drug event management	All the medication that is being given to the patient will be indicated. This will include the date on which the medication was started as well as all the dosages administered. This allows the pharmacist to have an overview of all the medication given to the patient and if the dosages were given according to the prescription. The patient should also be evaluated for untoward medication reactions, and all adverse drug events should be reported to the regulatory authority.	**Per episode**, each assessment per patient will constitute an individual episode and the patient should be reassessed per episode. Dispensing individual assessment from the patient's chart without a full assessment of the patient does **not** constitute an episode
Laboratory and Microbiological Assessments	This will include parameters like urea, creatinine and other electrolytes, blood elements, liver and kidney function tests, microbiology tests, and other tests as needed per patient case Urea, creatinine, and electrolytes need to be monitored daily as surgery and the use of certain medicines can affect these parameters; for example, surgery and cell damage can lead to hyperkalemia and the use of amphotericin can result in hypokalemia. Blood elements can be used to determine if the patient is, for example, anemic, if the patient has bone marrow suppression caused by certain drugs (e.g., chloramphenicol), or if a viral, bacterial, or parasitic infection is present. Liver and kidney function test is important to determine if any prescribed medication is contra-indicated for the patient or to monitor the adverse effects of the prescribed medication on the liver and kidney functions. Other parameters like C-reactive proteins can also be used as a marker of inflammation. Microbiology tests will be used to ensure that the patient receives specific antimicrobials to treat the specific infection to establish the rational use of antimicrobials.	**Per Patient Day:** This should be evaluated against the severity of the patient's condition; an episode can be constituted as one laboratory test which is evaluated against the patient's symptoms and vital signs.
Therapeutic Drug Monitoring	This involves individualization of dosage by maintaining plasma or blood drug concentrations within a target range (therapeutic range, therapeutic window). There are two major sources of variability between individual patients when discussing drug response. These variations are included in the relationship between: Dose and plasma concentration (pharmacokinetic variability), and Drug concentration at the receptor and the response (pharmacodynamic variability).	**Per Drug Per day:** Therapeutic drug monitoring starts when the drug is prescribed. It should first be determined if the dosage regimen is appropriate for the clinical condition being treated, considering the patient's clinical characteristics (age, weight, renal function, etc.) and concomitant drug therapy. Furthermore, when interpreting the concentration measurements, certain factors need to be considered, namely: Sampling time in relation to the dose The dosage history (i.e., whether the result represents steady state) The patient's response The desired clinical targets The above-mentioned information should then be used to adjust the dosage in order to achieve optimal response and to minimize toxicity.
Drug therapy assessment	The interventions should be classified according to the categories of drug-related problems as mentioned in the document	**Per patient per day:** document the interventions that are made on a day-to-day basis
Participation in multi-disciplinary team ward rounds or discussions and meetings	Attending and active participation in ward rounds and meetings	**Per episode:** Active participation in either a ward round or meeting constitutes an episode
Information on patient care or medicine use to other members of the multi-disciplinary team	Communication with other health care professionals or fellow pharmacists on patient-related issues or medicine use in patient care	**Per episode:** Each discussion relating to patient care or medicine use constitutes an episode

### Healthcare-Related Key Performance Indicators

Health care related key performance indicators are quantifiable measures of quality that can be used to track essential processes and specific outcomes. The measurement of processes by measurement of outcomes achieved by health care professionals’ activities, can be seen as the quality assessment of the professional’s practice. They can also assist to ensure accountability to protect patient safety as well as to inform other role-players in decision-making (Fernandes et al., 2015).

Pharmaceutical care related outcome measures to measure outcomes in specific disease-related programs where in-depth documentation with a data range were identified. However, when following the concept of pharmaceutical care as defined by Hepler and Strand, outcome-indicators were rather difficult to implement. Some of the pharmaceutical care indicators proposed is depicted in **Table 3** (Morak et al., 2010).

**Table 3:** Pharmaceutical care key performance indicators.

Indicator	Discussion	Outcome
Performance/Medicine substitution	Measures how often pharmacists substitute medicine in accordance with doctors to prevent drug interactions	Show the impact and success of pharmaceutical care
Hospital admission, frequency, and duration (after pharmacy interventions)	Possible in disease-specific pharmaceutical care programs with good documentation. Measurements could be taken before and after the pharmacy-led intervention.	Show an increase/reduction of hospital stay after pharmacist-led intervention
Number of pharmacy-led interventions	Relatively easy to measure. However, there is large room for interpretation. Depth of intervention could pose problematic.	Show the number of times a pharmacist intervenes in drug therapy
Number of drug-related problems / medication errors	Classical indicator with regard to pharmaceutical care; however, the possibility of measurement depends on the vigilance systems in different settings.	Show possible reduction of drug-related problems/ medication errors in a disease-specific pharmaceutical care. Can be performed with a rather small group of people.
Patient satisfaction	Regularly evaluated together with pharmaceutical care programs.	Difficulty in interpretation of this indicator, because often not subjective.
Regular customers Trust Patient-pharmacist relationship Prescriber-pharmacist relationship	Considered as very subjective; therefore, it is difficult to measure and compare it between practitioners and settings.	Show the relationship of pharmacist with patients, customers, or prescribers.
Process indicators (on key elements of pharmaceutical care, e.g., counselling, documentation)	Proposed indicators are questions on the process: “Is electronic documentation available?” “Is clinical pharmacy implemented?” “Are there indications having intensive programs?”	Questions answered easily, and can show the level of presence of pharmaceutical care.
Health status indicators, e.g., morbidity rates	Easily measurable and standard indicators in many health systems. Interpretation can be problematic, as it is difficult to attribute an improvement in health to pharmaceutical care only but may include a number of many other factors.	May show improvement over period of time.

Adapted from Morak et al. (2010).

## Conclusion

Adoption of practice guidelines may strengthen clinical pharmacy practice in South Africa, and ultimately improve the quality of care for patients. Since clinical pharmacy as a specialization cannot be registered at the pharmacist regulatory body in South Africa yet, official clinical pharmacists have not been appointed in the allocated posts. The goal of the practice guidelines is to ensure that clinical pharmacists possess the core competencies necessary to contribute to a quality standard of clinical pharmacy and therefor optimize medication use in South Africa.

Clinical pharmacist individuals, postgraduate training programs at universities, private health care institutions as well as the NDoH need to commit to achieve and maintain these competencies and practice guidelines to ensure benefit to patients. The scope of practice of clinical pharmacists has been published by the regulatory body, however, private hospital groups has developed job descriptions for clinical pharmacists with key performance indicators. A need for a standardized standard of practice is evident.

## Author Contributions

All authors listed have made a substantial, direct, and intellectual contribution to the work and approved it for publication.

## Conflict of Interest

The authors declare that the research was conducted in the absence of any commercial or financial relationships that could be construed as a potential conflict of interest.

## References

[B1] American College of Clinical Pharmacy (ACCP) (2014). Standards of Practice for Clinical Pharmacists. Pharmacotherapy 34 (8), 794–797. 10.1002/phar.1438 25112523

[B2] American College of Clinical Pharmacy (ACCP) (2017). Comprehensive medication management in team-based care. American College of Clinical Pharmacy World Headquarter Available at: https://www.accp.com/docs/positions/misc/CMM%20Brief.pdf. Assessed 18 September 2018.

[B3] American Society of Hospital Pharmacists (1992). Clinical Skills Program. Advancing Pharmaceutical Care (Bethesda: American Journal of Health-System Pharmacy). Available at: www.ashp.org.com. Assessed 23 June 2012.

[B4] Berg M. J. (2001). Postgraduate Pharmacy Education in Developing Countries. [Conference Report]. 61st International Congress of FIP, Singapore, September 1-6Medscape Pharmacists 2(2). 2001. Available at: http://www.medscape.com/viewarticle/408585. Viewed 26 June 2014.

[B5] Dowse R. Kanfer I. (1987). Clinical Pharmacy: What’s it all about? Part I: Development and Practice of Clinical Pharmacy. South Afr. Pharm. J. 54 (7), 181–183.

[B6] Fernandes O. Gorman S. K. Slavik R. S. Semchuck W. M. Shalansky S. Bussieres J. (2015). Development of Clinical Pharmacy Key Performance Indicators for Hospital Pharmacists Using a Modified Delphi Approach. Ann. Pharmacother. 49 (6), 656–669. 10.1177/1060028015577445 25780250

[B7] Gilbert L. (1998). Pharmacy’s Attempts to Extend its Roles: A Case Study in South Africa. Soc. Sci. Med. 47 (2), 153–164. 10.1016/S0277-9536(98)00022-7 9720635

[B8] Hepler C. D. Strand L. M. (1990). Opportunities and Responsibilities in Pharmaceutical care. Am. J. Hosp. Pharm. 47, 533–543. 10.1093/ajhp/47.3.533 2316538

[B9] Hepler D. (2004). Clinical Pharmacy, Pharmaceutical Care and the Quality of Drug Therapy. Pharmacotherapy 24 (11), 1491–1498. 10.1592/phco.24.16.1491.50950 15537552

[B10] Matshotyana K. (2009). Job Satisfaction of Public Sector Pharmacists in the Nelson Mandela Metropole. Unpublished Masters Dissertation in Health and Welfare Management (Port Elizabeth: Nelson Mandela Metropolitan University).

[B11] Morak S. Vogler S. Walser S. Kilstra N. (2010). Understanding the Pharmaceutical Care Concept and Applying it in Practice. Ed. Gesundheit Osterreich G. (Vienna: Australian Federal Ministry of Health).

[B12] Murphy J. E. Nappi J. M. Bosso J. A. Saseen J. J. Hemstreet B. A. Halloran M. A. (2006). American College of Clinical Pharmacy’s Vision of the Future: Postgraduate pharmacy Residency Training as a Prerequisite for Direct Patient Care Practice. Pharmacotherapy 26 (5), 722–733. 10.1592/phco.26.5.722 16637798

[B13] National Department of Health (2011). National core Standards for health Establishments in South Africa, Available at: rhap.org.za/wp-content/uploads/2014/05/National-Core-Standards-2011-1.pdf.

[B14] National Department of Health (2015). National Health Insurance for South Africa: Towards universal health coverage, Available at: http://www.health.gov.za/index.php/component/phocadownload/category/383. Assessed 6 November 2018.

[B15] Padarath A. Chamberlain C. McCoy D. Ntuli A. Rowson M. Loewenson R. (2003). Health Personnel in Southern Africa: Confronting Maldistribution and Brain Drain (Health Systems Trust (South Africa) and MEDACT (UK): Regional Network for Equity in Health in Southern Africa (EQUINET)). EQUINET discussion paper number 3.

[B16] Saseen J. J. Ripley T. L. Bondi D. Burke J. M. Cohen L. J. McBane S. (2017). ACCP Clinical Pharmacist Competencies. Pharmacotherapy 37 (5), 630–636. 10.1002/phar.1923 28464300

[B17] Schellack N. Gous A. G. S. (2010). An Assessment of the Need for Pharmaceutical Care in an NICU in South Africa (LAP Lambert Academic Publishing GmbH & Co.KG). Available at: https://www.lap-publishing.com

[B18] South African Pharmacy Council (SAPC) (2010). Good Pharmacy Practice Manual. 4th ed. (Copyright Office of the Registrar, SAPC: Compiled and Edited by the Office of the Registrar).

[B19] Summers R. S. Haavik C. Summers B. Moola F. Lowes M. Enslin G. (2001). Pharmaceutical Education in the South African Multicultural Society. Am. J. Pharm. Educ. 65 (2), 150–154.

[B20] Summers B. (1991). Mirror to Hospital Pharmacy in Southern Africa (Pietermaritzburg: The Natal Witness Printing and Publishing Company).

